# Induction of Th1Immune responses following laser ablation in a murine model of colorectal liver metastases

**DOI:** 10.1186/1479-5876-9-83

**Published:** 2011-05-29

**Authors:** Wen Xu Lin, Theodora Fifis, Caterina Malcontenti-Wilson, Mehrdad Nikfarjam, Vijayaragavan Muralidharan, Linh Nguyen, Christopher Christophi

**Affiliations:** 1 Department of Surgery, University of Melbourne, Austin Hospital, Heidelberg, Australia

## Abstract

**Background:**

Preliminary experimental studies have suggested that the in situ destruction of tumor tissue by local laser ablation (LA) may also stimulate host immunity against cancer. We investigated local and systemic induction of immune responses after laser ablation in the setting of residual tumor.

**Methods:**

A murine colorectal cancer (CRC) liver metastasis model was used. Selected tumors of liver CRC bearing mice and livers of mice without tumor induction were treated with LA. Liver and tumor tissues from the ablation sites and from distant sites were collected at various time points following LA and changes in CD3+ T cells and Kupffer cells (F4/80 marker) infiltration and the expression of interferon gamma (IFNγ) were investigated by immunohistochemistry and ELISpot. Base line levels of CD3+ T cells and Kupffer cells were established in untreated mice.

**Results:**

The presence of tumor induced significant accumulation of CD3+ T cells and Kupffer cells at the tumor-host interface, within the tumor vascular lakes and increased their baseline concentration within the liver parenchyma. LA of the *liver *induced accumulation of CD3+ T-cells and Kupffer cells at the site of injury and systemic induction of immune responses as discerned by the presence of IFNγ secreting splenocytes. LA of liver *tumors *induced significant increase of CD3+ T-cells at site of injury, within normal liver parenchyma, and the tumor-host interface of both ablated and distant tumors. In contrast Kupffer cells only accumulated in ablated tumors and the liver parenchyma but not in distant tumors. IFNγ expression increased significantly in ablated tumors and showed an increasing trend in distant tumors.

**Conclusion:**

Laser ablation in addition to local tumor destruction induces local and systemic Th1 type immune responses which may play a significant role in inhibiting tumor recurrence from residual micrometastases or circulating tumor cells.

## Background

Colorectal cancer (CRC) is the most common solid organ cancer across both genders and the third most common cause of cancer related deaths [1]. More than 50% of patients with CRC develop liver metastases (CRCLM) which is the leading cause of death in this population. Surgical resection is the only potential curative option. The spatial distribution of metastases, presence of extra hepatic disease, potential residual liver volume and function as well as the general health of the patient are the main factors that limit the surgical option to approximately 10-25% of patients [2,3]. Advances in systemic therapies have progressively increased the potential for surgical intervention by down staging hepatic metastases in a small subset of patients [4]. Despite successful surgery, the majority of patients develop disease recurrence most frequently in the liver.

Local thermal ablation was developed to increase the therapeutic options for patients with liver metastases [5,6]. This involves the application of laser, radiofrequency or microwave energy to the tumor. The particular energy in each case is converted into heat that leads to tumor destruction by coagulative necrosis. The aim is to extend the necrosis into a rim of normal tissue parenchyma surrounding the tumor for total tumor destruction [7-9]. When applied as a minimally invasive technique, thermal ablation has a number of potential advantages including significantly lower morbidity, minimal destruction of normal liver tissue and transient changes in liver function enzymes, leading to a lesser regenerative response and the ability for repeated application [10-13].

Early clinical comparisons between resection and thermal ablation suggested that thermal ablation is associated with a less favourable outcome [5]. Results from experimental animal studies however suggest that thermal ablation of metastatic liver tumors is associated with reduced incidence of tumor growth and metastasis compared to resection. Additionally a positive effect on host immune response has been reported following thermal ablation of tumors where the ablated tumor antigens appear to behave like a tumor vaccine [14-16].

This study investigated immune responses in mice with CRC liver metastases following LA of selected tumors. In particular, it focused on changes of Kupffer cells (or tumor infiltrating macrophages; TAMs) and CD3+ T cells representing innate and adaptive immunity respectively and on IFNγ expression which is associated with Th1 protective immune responses in cancer [17]. The experimental plan was designed to investigate if protective immune responses occur in a scenario reflecting clinical application of LA, where residual micrometastases or tumor at the margins of an ablation site remain after treatment.

## Methods

### Animals

Six to eight week old male CBA mice (Laboratory Animal services, University of Adelaide, South Australia) were used in all experiments. Mice were maintained in standard cages with access to irradiated food and water ad libitum, and exposed to a twelve hour light/dark cycle. All procedures were implemented in accordance with the guidelines of the Austin Health Animal Ethics Committee.

### Experimental model of CRC liver metastases

The primary cell line MoCR was derived from a dimethyl hydrazine (DMH)-induced primary colon carcinoma in the CBA mouse and maintained *in vivo *by serial passage in the flanks of CBA mice [18]. For passage and experimentation, tumors grown subcutaneously were teased, passed through a filter, treated with EDTA and washed in PBS to make a single cell suspension. Liver metastases were induced by an intrasplenic injection of 5 × 10^4 ^tumor cells prior to splenectomy as reported previously [18]. In this model, liver metastases are fully established by 21 days following tumor induction.

### Laser Ablation Treatment

Twenty-one days after tumor induction animals were used for LA study. Similarly located intra-parenchymal tumors of 7 mm diameter were chosen for sub-total laser ablation and was performed as described previously [19]. Briefly a Neodymium Yttrium-Aluminium-Garnet (Nd:YAG-wavelength of 1064 nm) laser (Dornier medilas fibertom 4100 Medizintechnik GmbH, Munchen) was used. Animals were anaesthetized and a bilateral sub-costal incision was performed to allow full exposure of the liver. A 400 μm bare tip optical quartz fibre delivered laser energy, applying 100J of power per tumor (50 seconds at 2 Watts). The treatment parameters were chosen based on our previous extensive studies where the nature and extent of injury including temperature profiles were examined [19-21]. Average tissue temperatures reach 65°C adjacent to the fibre site without causing tissue charring. Higher power settings in this animal model generally produce charring. This setting in tumor tissue produces incomplete necrosis that does not extend into the liver. For endpoints other than 0 the treated tumors were marked with special dye (Davidson Tissue Marking System, Bradley Products, Grale Scientific, Melbourne, Australia), the abdomen was closed and animals recovered.

### Tissue Sample Collection

At each endpoint after LA treatment, mice were anesthetized and their liver was excised. The two ablated or sham treated (no activation of the probe) tumors were identified and then immediately dissected from the liver together with surrounding liver tissue. Samples of liver tissue and untreated tumors were also collected. All specimens were fixed in formalin for 48 hours and processed for immunohistochemistry.

### Experimental Design

Three study groups were used: The *first *study aimed to establish the baseline distribution of T cells and Kupffer cells in tumor bearing livers and consisted of two groups of mice. The experimental group was induced with metastatic tumor cells 21 days prior to tissue collection and controls consisted of a group of mice from the same cohort but not induced with tumor. The *second *study investigated temporal changes in the distribution of T cells, Kupffer cells and IFNγ expression, when non tumor bearing animals were treated with TA in the liver tissue and compared to baseline controls (shams: liver not treated with TA). The *third *study investigated temporal changes in the distribution of T cells, Kupffer cells and IFNγ expression in liver and metastatic tumor tissues following TA treatment of two selected tumors. The results were compared to baseline controls (day 21 post tumor induction and day 0 post TA treatment).

### Immunohistochemistry

Formalin fixed paraffin embedded 4-μm-thick sections of the tissues were deparaffinized and rehydrated using standard techniques. Endogenous peroxidases were blocked by incubation in 3% peroxide in methanol for 10 minutes. Antigen retrieval was achieved by incubation in Proteinase K in a 37°C oven for 20 minutes, followed by a cooling down period of 10 minutes at room temperature (RT). Normal goat serum (20%) was used to block non specific binding. Commercially available primary antibodies used for staining (CD3; rabbit anti-human CD3+ polyclonal A0452, Dakocytomation, Denmark at 0.6 μg/ml, IFNγ; rat anti-mouse IFNγ monoclonal 3321-3-1000, Mabtech, Australia, at 1 μg/ml, Kupffer cell staining; rat anti mouse F4/80 monoclonal antibody, ATCC no. HB-198, culture supernatant at 1:50 dilution). Negative controls were incubated with the respective non immune antibody isotypes at the same concentration as the primary antibody. Sections were incubated with primary antibodies overnight at 4°C. Sections treated with the rat antibodies were treated with a rabbit anti-rat linker antibody before treatment with a polymer based detection kit containing goat anti-rabbit immunoglobulins (IgG) linked to horseradish peroxidase (HRP) (Envision Plus, Dako, Australia). Each incubation step was followed by two five minute washes with PBS + 0.05% Tween 20. Positive staining was visualized using diaminobenzidine (DAB) as a substrate.

### ELISpot assay

Mouse spleens were collected from LA treated and sham LA treated mice and the spleen cells from each group were pooled. Spleen cells (10^6 ^per well in 100 μL RPMI complete medium) were incubated without stimulation for 18 h in 96-well plates (MAIPS; Millipore, Australia) pre-coated with host species anti-murine IFNγ (clone R4, American Type Culture Collection, Manassas, VA). Triplicate wells were set up for each condition. After washing wells with PBS, secreted cytokine was detected with biotinylated anti-murine IFNγ (MAb XMG.21-biotin; Pharmingen, Australia) followed by extravidin-alkaline phosphatase at 100 μg/mL (Sigma). Spots of activity were detected with a colorimetric alkaline phosphatase kit (Bio-Rad, Hercules, California, USA) and counted using a plate reader (AID GmbH, Germany) with AID ELISpot software Version 3.0. Data are presented as mean spot-forming units (SFU) per million cells ± standard error of the mean (SEM).

### Quantification of CD3+ T cell and Kupffer cell staining

All sections were examined using a digital microscope system (Coolscope, Nikon Corporation, Chiyokd-ku, Tokyo, Japan). Areas of interest were identified and photomicrographs for each region were captured for enumeration of lymphocytes within (1) tumor-host interface (treated and untreated distant tumors), (2) LA injury front and (3) distant normal liver away from the ablation sites. Images were coded and analyzed using an image analysis program in a blinded manner (Image-Pro Plus Version 4.5.1, Media Cybernetics, USA). Counts were expressed as the number of positive cells per mm^2 ^of tissue. Alternatively positive stained areas were calculated using Image-Pro Plus software and expressed as arbitrary units.

### Semi-quantitative analysis of IFNγ

Areas of interest were identified using a light microscope (Olympus BH2, Japan) at a magnification of 125x. The entire margin of treated normal liver, tumor host interface of treated/untreated tumor and normal liver tissues were examined. Scoring criteria was used to estimate the amount and intensity of staining seen in each sample. The grading system used was: as: 0: no staining 1: faint staining; 2: small amount or weak staining; 3: moderate staining; 4: abundant or strong staining; 5: Abundant or very strong staining. Means for each group were determined using the individual scores from each sample.

### Statistical assessment

Statistical analyses were performed using SPSS program (Statistical Package for the Social Sciences™, version 10, Chicago, Illinois, USA). All data was expressed as the mean ± standard error of the mean unless otherwise specified. Data was tested for normality using detrended Q-Q plots, descriptive statistics such as skewness and kurtosis and the Kolgomorov-Smironov test prior to statistical analysis. Differences between groups were assessed by non parametric Kruskall Wallis followed by Mann Whitney U tests or parametric ANOVA followed by Tukey post hoc analysis as appropriate. A P value of 0.05 or less was regarded as statistically significant.

## Results

### Tumor induces accumulation of CD3+ T cells and Kupffer cells in liver and tumor tissues

**Figure 1:**
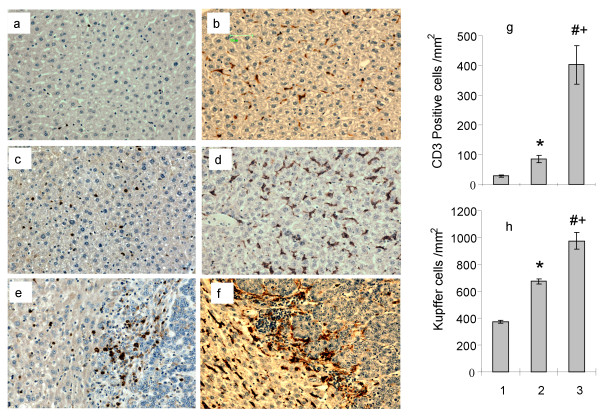
**Tumor induced accumulation of CD3+ T cells and Kupffer cells**. Liver sections from mice with CRC liver metastases 21 days post tumor induction (n >5) and from naive mice (n = 5) were immunostained for Kupffer cells (F4/80 antibody) and CD3+ T cells (anti-CD3+). Panels a and b: liver parenchyma from naïve mice stained with anti-CD3+ and F4/80 respectively. Panels c and d: liver parenchyma from tumor bearing mice stained with anti-CD3+ and F4/80 respectively Panels e and f: tumor sections depicting the tumor-liver parenchyma interface stained with anti-CD3+ and F4/80 respectively. Original magnification 200x Panels g and h: concentration of CD3+ T cells and Kupffer cells expressed as mean number of cells/mm^2 ^± SEM. 1, liver tissues from naïve animals; 2, liver tissues from tumor induced animals; 3, tissues from tumor-liver parenchyma interface. (CD3+ T; * P < 0.002 compared to naive, # P = 0.003 compared to naive, + P < 0.003 compared to tumor bearing liver.) (Kupffer cells; * P < 0.0003 compared to naive, # P < 0.0003 compared to naive, + P < 0.0003 compared to tumor bearing liver.)

The presence of liver metastases increased the concentration of both CD3+ T cells and Kupffer cells in the liver parenchyma as seen in Figure 1 (panels a and c CD3+ immunostained sections, panels b and d F4/80 immunostained sections. Tumor bearing CD3+ cell count; 85.2 ± 12.1 cell/mm^2 ^vs. naive 28.3 ± 2.8 cell/mm^2^, P < 0.002 and Kupffer cell count; 673.1 ± 39.6 cell/mm^2 ^vs. naive 370.6 ± 10.6 cell/mm^2^, P < 0.0003, panels g and h respectively). Accumulation of both cell types was observed in the tumor tissues especially at the tumor/host interface and within the vascular lakes (Figure 1 panel e for CD3+ T cells and f for Kupffer cells). The concentration of both cell types at the tumor host interface were significantly higher than those in liver parenchyma of naïve animals (Figure 1 panels g and h, CD3+ cells; 402.2 ± 64.64.9 cell/mm^2 ^vs 28.3 ± 2.8 cell/mm^2^, P = 0.003 and Kupffer cells: 975.6 ± 61.9 vs 370.2 ± 10.6 cells/mm^2^, P < 0.0003) and also significantly higher than the concentration in tumor bearing liver parenchyma (Figure 1 panels g and h, CD3+ cells: 402.2 ± 64.64.9 vs. 85.2 ± 12.1 cell/mm^2^, P < 0.003 and Kupffer cells: 975.6 ± 61.9 vs 673.1 ± 39.6 cell/mm^2^, P < 0.0003). Therefore, these results show that tumor presence results in significant accumulation of CD3+ T cells and Kupffer cells not only within the tumors but also in the liver parenchyma.

### Laser ablation of liver tissue induces local and systemic immune responses

**Figure 2:**
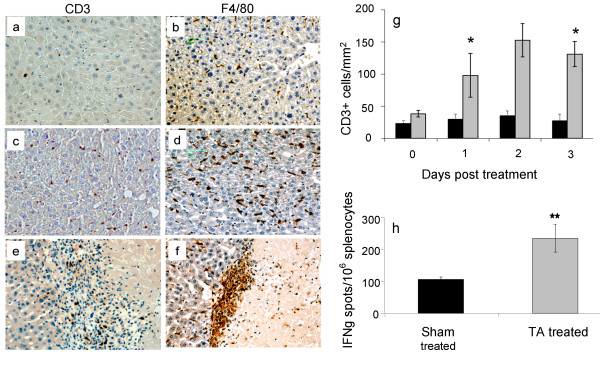
**Immune responses following LA treatment of liver tissue**. Groups of mice (n >5) had LA treatment in the liver and tissue was collected at various time points post ablation as indicated. Control mice had sham LA performed (LA probe was not activated). Panels a and b, sham treated liver sections at three days post treatment. Panels c and d, liver sections distant from the site of ablation at three days post treatment. Panels e and f, ablated liver sections 3 days post treatment depicting the injury front. Original magnification 200x. Panel g, temporal changes in CD3+ T cells at the injury front following LA treatment expressed as mean number of cells/mm^2 ^± SEM, * P < 0.05, compared to control. Panel h, enumeration of IFNγ secreting splenocytes at 3 days post liver LA treatment, expressed as mean number of spots/million splenocytes ± SEM, ** P < 0.001, compared to control.

This study examined local and systemic immunological effects of LA treatment on livers from animals with no tumors. LA treatment resulted in the accumulation of CD3+ T cells and Kupffer cells at the site of injury following treatment compared to sham controls. (Figure 2 panels a and e for CD3+ cells panels b and f for Kupffer cells). CD3+ T cell accumulation at the injury site persisted over the next three days with the peak occurring on day 2 following LA. (Figure 2g CD3+ increase compared to sham treated liver. P values: Immediate: 0.068; day 1: 0.046; day 2: 0.053; day 3: 0.034, Mann Whitney U tests). In an earlier study, we demonstrated that Kupffer cell accumulation displayed a biphasic increase, with an initial peak followed by a decrease over the next two days, and then another peak at days three to five [19]. In this respect the Kupffer cell kinetics were different to those of CD3+ T cells, where a single peak was observed in the current study. Increases in both Kupffer and CD3+ T cell concentration after ablation were also observed within the distant uninjured parenchyma (Figure 2c and figure 2d respectively and Additional file 1, Figure S1). LA treatment of the liver also induced a systemic immune response. Significantly greater number of splenocytes from treated animals secreted IFNγ at three days after LA treatment compared to controls (Figure 2h; 235 ± 43.5 vs 106.3 ± 8.0 IFNγ secreting cells per million splenocytes respectively, P < 0.001). These results represent *in-vivo *stimulation as the splenocytes were not further stimulated during the ELISpot assay, indicating that LA treatment induces immune responses not only locally but also systemically even in the absence of tumor.

### Laser ablation of selected tumors induces concentration and distribution changes of CD3+ T cells in tumor and liver tissues

**Figure 3:**
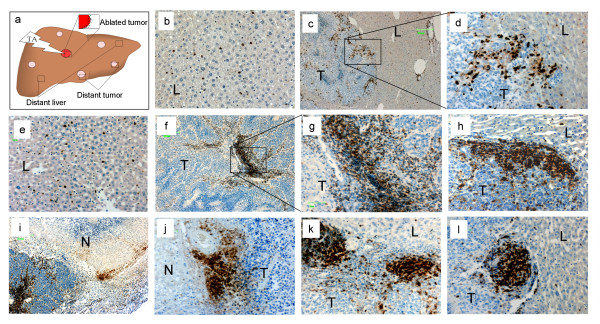
**Changes in CD3+ T cell frequency and distribution following LA treatment of selected liver metastases**. Groups of mice (n >5) had two tumors LA treated and tissues were collected at various time points post ablation from areas indicated in the diagram of panel a. Control mice had sham LA performed (LA probe was not activated). Panels b-d are CD3+ stained tissues from control animals; b liver parenchyma, c tumor host interface and d an enlarged section of c as shown in the rectangle. Panel e depicts a section of liver parenchyma from an LA treated animal immediately following ablation. Panels f-l are CD3+ stained tumor sections from LA treated animals collected at day 2 post treatment. Panels f and g depict CD3+ staining of a vascular lake in a distant tumor; g is an enlarged section of panel f as shown in the rectangle. Panel h depicts a section of distant tumor host interface Panels i and j depict sections of the ablated tumor injury front. Panels k and l depict sections of the ablated tumor host interface. Panels c, f and i original magnification 50x, all other panels original magnification 200x. L, liver; T, tumor; N, necrotic tissue within the injury front.

Changes in concentration and distribution of CD3+ T cells in tumor and liver tissues (Figure 3a) following LA of two tumors were investigated at various time points post treatment and compared to sham treated mice. Sham treatment did not significantly change the frequency or distribution of CD3+ T cells in any of the liver tissues compared to untreated animals. Furthermore there was no significant temporal change during the different time points post sham treatment. In contrast, LA treatment produced an immediate increase in CD3+ T cell concentration in the liver parenchyma above the sham values (Figure 3e compared to Figure 3b). Immediate increases were also seen at the tumor host interface of ablated and distant tumors, at the ablation injury front and within vascular lakes of ablated and distant tumors. These increases persisted at high levels compared to shams at all time points (Figure 3 panels f-h for distant tumors and panels i-l for ablated tumors). CD3+ T cells were not uniformly distributed however, with patches of very high numbers observed at the injury front (Figure 3 panels i and j) and at the tumor host interface (Figure 3 panels k and l) while in neighbouring regions accumulation was a lot less prevalent. Similarly some vascular lakes within distant tumors were observed to be densely populated with CD3+ T cells while others were not. In general the most consistent accumulation was observed at the tumor-host interface of both ablated and distant tumors.

**Figure 4:**
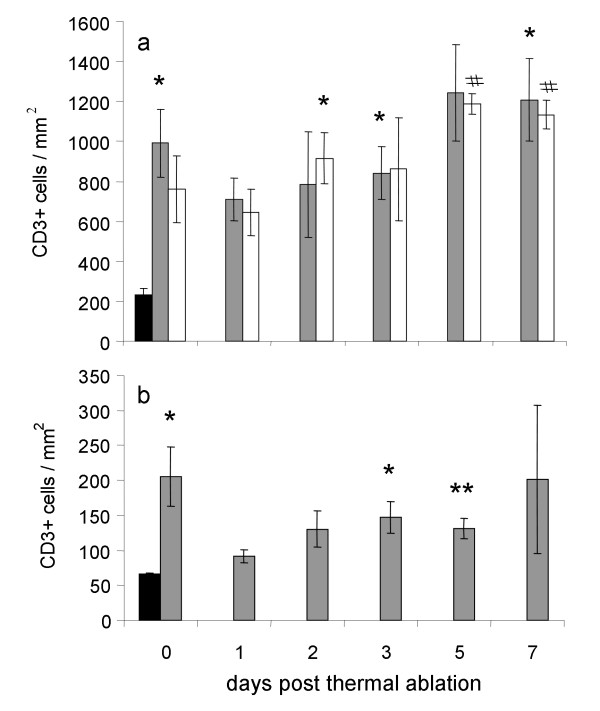
**Temporal changes in CD3+ T cells following LA treatment of selected liver metastases**. Groups of mice (n >5) had two tumors LA treated and tissues were collected at various time points post ablation. Control mice had sham LA performed (LA probe was not activated). Panel a temporal changes in CD3+ T cells at the tumor host interface; grey bars = LA treated tumors, white bars = distant tumors and black bars = sham controls. Panel b temporal changes in CD3+ T cells in the liver parenchyma post LA treatment. Black bars = sham controls, grey bars = LA treated. Results are expressed as mean number of cells/mm^2 ^± SEM. * and # P < 0.05, * * P < 0.01 compared to controls.

Temporal changes in CD3+ T cells at the tumor-host interface of ablated and distant tumors were biphasic with an initial rise followed by a small decrease and then a second higher peak occurring between day 3 to 7 for both the ablated and distant tumor-host interface (Figure 4). Infiltration of CD3+ T cells at the tumor host interface of LA treated tumors was increased at all time points post treatment compared to sham operated tumor-host interface (P values: 0.050; 0.061; 0.066; 0.050; 0.060 and 0.034; for time points 0,1,2,3,5 and 7 days respectively; Figure 4a. grey bars). Similarly infiltration of CD3+ T cells increased at tumor-host interface in distant tumors of LA treated animals compared to equivalent tissues of sham treated animals (P values: 0.119; 0.098; 0.026; 0.184; 0.000 and 0.003; for time points 0, 1, 2, 3, 5 and 7 respectively; Figure 4a. white bars).

Temporal biphasic changes of CD3+ T cell numbers were seen within the liver parenchyma distant from the ablation sites Figure 4b. These changes showed an immediate peak which was then followed by a second peak, with significant differences seen in CD3+ T cell numbers between the treated and sham groups (P values: Immediate: 0.050, Day 1: 0.086; Day 2: 0.086; Day 3: 0.043; Day 5: 0.008; Day 7: 0.433, t tests) after LA treatment, suggesting systemic trafficking of these cells.

### Laser ablation of specific tumors induces increased IFNγ expression in tumor tissues

**Figure 5:**
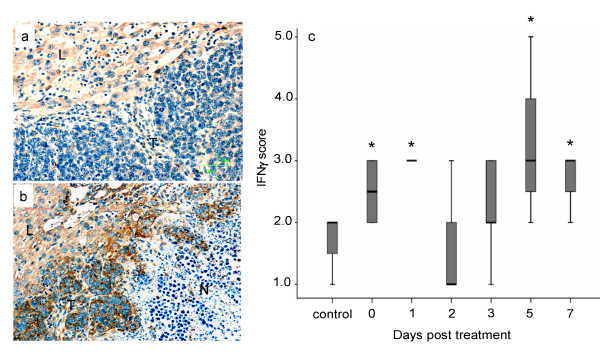
**Temporal changes in IFN**γ **expression following LA treatment of selected liver metastases**. Groups of mice (n >5) had two tumors LA treated and tissues were collected at various time points post ablation. Control mice had sham TA performed (LA probe was not activated). Panels a and b tumor sections from control (sham ablated) and LA treated animals at day 5 post treatment respectively, stained with rat anti IFNγ monoclonal antibody. L; liver, T; tumor, N; necrotic area at the LA injury front. Panel c; temporal changes in IFNγ expression at the tumor host interface and injury front expressed as mean intensity score ± SEM. * P < 0.05 compared to sham control.

Tissues were collected as described in Figure 3a and immunostaining for IFNγ was performed. The expression of IFNγ appears diffuse and generalised rather than localised within cells and therefore a scoring technique was used. Immediately following treatment, there was increase in staining at the tumor host interface when compared to sham treatment (Figure 5 a and 5b). Following LA treatment, IFNγ expression over time displayed a biphasic pattern (Figure 5c) with an initial peak at day 1 followed by a second peak between days 3 and 7 similar to that seen for CD3+ T cells (P values Day 0: 0.171, Day 1: 0.022; Day 2: 0.703; Day 3: 0.210; Day 5: 0.044; Day 7: 0.040, compared to sham treated, Mann Whitney U tests). IFNγ expression also increased at distant tumor host interface; however the increases did not reach significance levels (result not shown).

### Changes in concentration and distribution of Kupffer cells in tumor and liver tissues following laser ablation of tumors

**Figure 6:**
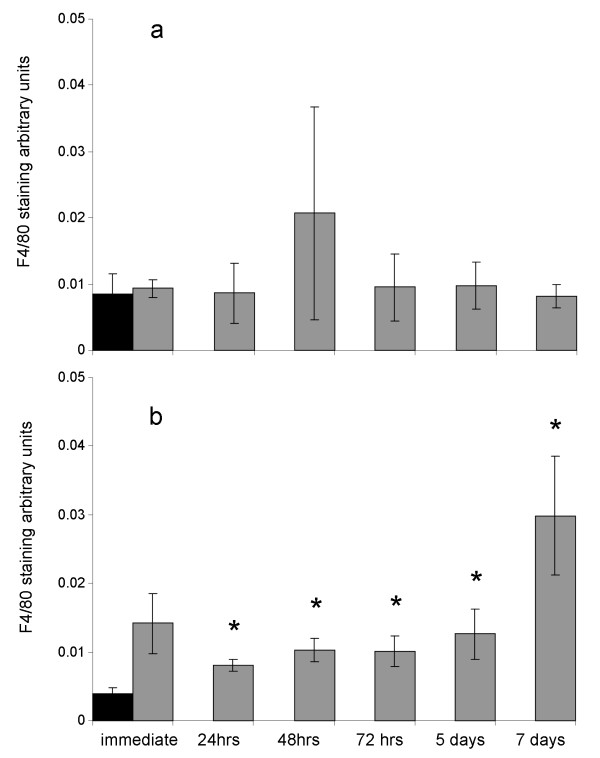
**Temporal changes in Kupffer cells following LA treatment of selected liver metastases**. Groups of mice (n >5) had two tumors LA treated and tissues were collected at various time points post ablation. Control mice had sham LA performed (LA probe was not activated). F4/80 positive areas were calculated using image pro plus software. Values are expressed as mean arbitrary units ± SEM. Panel a; black bar, Kupffer cells at the tumor host interface distant from sham treated tumors; grey bars, Kupffer cells at the tumor host interface distant from ablated tumors. P > 0.05 compared to sham control at all time points. Panel b; black bar, F4/80 staining in liver tissue of sham treated mice; grey bars, F4/80 staining in liver tissue distant from ablated tumor site. * P < 0.05 compared to sham control.

In a previous study we have shown that Kupffer cell numbers significantly reduced at site of the tumor ablation injury during the first two days following treatment and then significantly increased, peaking on day 3 but remaining significantly elevated compared to untreated control for all further time points tested [19]. In the present study we examined temporal changes of KC at the margins of untreated tumors distant to the ablation site. There was no significant difference between sham ablated and ablated groups (Figure 6a), however significant increases were seen in the liver parenchyma distant to ablation site in the LA treated compared to sham treated animals (Figure 6b), suggesting systemic trafficking of these cells.

## Discussion

Thermal ablation has evolved as a significant minimally invasive treatment for unresectable CRC liver metastases as well as an adjunct to liver resection [22]. Accumulating evidence suggests that *in situ *tumor destruction by thermal ablation may also stimulate local and systemic anti-tumor immunity, with the potential to eliminate not only treated tumors, but also residual micrometastases which normally give rise to tumor recurrence; reviewed by Gravante et al [16]. In previous studies we have shown that LA destroys tumor or liver tissue by generating immediate focal necrosis followed by a marked inflammatory response and progressive increase in the area of injury [8]. We have also demonstrated significant accumulation of Kupffer cells and increased expression of HSP70 at the injury front persisting for a number of days following LA treatment [19]. In the present study we demonstrate T-cell accumulation not only at the LA injury site, but also within liver parenchyma and tumor/host interface of both ablated tumors and residual tumors distant from the site of ablation. In contrast Kupffer cells only accumulated in ablated tumors and the liver parenchyma but not in distant tumors. IFNγ expression increased significantly in ablated tumors and showed an increasing trend in tumors distant from the ablation site. In addition significantly more splenocytes from liver ablated animals secreted IFNγ compared to controls.

In clinical studies, thermal ablation of tumors has been shown to result in early systemic inflammation, the induction and systemic trafficking of specific anti-tumor T-cell responses involving CD4+ and CD8+ T cells [23,24] and a generalized adjuvant effect that also involved the activation of natural killer cells [25,26].

In experimental studies, total tumor destruction by thermal ablation protected animals from further tumor challenge [27] while partial tumor removal by thermal ablation resulted in significant residual tumor inhibition and systemic tumor specific CD4+ and CD8+ T-cell induction compared to resection [28]. The mechanisms by which thermal ablation activates the immune system are not clear at this stage, accumulating evidence however suggest the involvement of both the innate and the adaptive immune systems and their cytokines [16].

Our results indicate that presence of tumor alters the molecular environment of the liver in ways that attract accumulation of immune cells (CD3 T cells and Kupffer cells). Infiltration of Kupffer cells (or TAMs) into tumors has been reported in many other studies. Macrophages activated in the classical pathway (M1) favoring a Th1 immune response (IFNγ, NO, TNFα, IL-1 and IL-12 secretion) are associated with tumoricidal functions. Macrophages infiltrating the tumor microenvironment however are usually activated along the M2 pathway promoting Th2 type immune responses [29] and tumor progression by releasing proangiogenic cytokines and growth factors (VEGF, IL-8, b-FGF) and matrix metalloproteases (MMPs) that digest the tumor basement membrane, facilitating tumor metastasis [30].

Infiltration and accumulation of CD3+ T cells within colorectal and other tumors has also been reported in several other studies. The significance of this infiltration is controversial. Early studies associate it with a favourable outcome [31,32]. More recent studies however indicate that T cell infiltration in solid tumors are at best ineffectual in controlling tumor growth and most often contribute to tumor progression by enabling the neutralisation of immune responses [33]. The tumor microenvironment subverts the immune response in a variety of ways to support tumor growth. All CD3+ T cell subtypes have been shown to be capable of promoting tumor progression, either through altered cytokine production such as IL-1, IL-4, TGF-β and IL-10 [34-36] or through cell-cell contact after being converted into FoxP3 regulatory T cells by the influence of tumor stroma derived immunosuppressive factors such as PGE2, TGF-β or IDO by-products [37,38]. Thermal ablation studies suggest that the treatment induces protective Th1 immune responses to counteract the immunosuppressive tumor microenvironment. Antigens from thermally ablated hepatocellular carcinoma induced superior stimulation of *in vitro *immune responses than untreated tumor antigens [39] and vaccination with antigens from thermally treated tumors prior to thermal ablation enhanced the treatment outcome [40]. This is most likely achieved by the upregulation of HSP proteins including HSP70 that we and others have shown to occur after LA treatment [19]. HSP70, a stress induced molecular chaperone, has a dual role in inducing a Th1 anti-tumor response. HSP70 acts as a general adjuvant, signaling through toll-like receptor 4 (TLR-4) [41] resulting in the maturation and activation of dendritic cells (DCs). Maturation of DCs is required to efficiently present antigenic peptides for protective immune responses. HSP70 also forms complexes with all the tumor antigens so it also induces tumor specific immune responses by delivering specific antigens to DCs [42]. Maturation of dendritic cells and efficient antigen presentation results in a Th1 immune response capable of overcoming the tumor immunosuppressive environment. It was shown that a Th1 response and upregulation of IFNγ is required for the prevention of tumor establishment or the elimination of already established tumors [17]. The presence of Th1 activated T cells is an independent prognostic marker for patient survival [43].

Upregulation of the Th1 pathway cytokines IL-12 and/or IFNγ within the tumor resulted in tumor killing [44] and directly inhibited tumor angiogenesis [45]. In the current study we demonstrated local and systemic upregulation of IFNγ and the accumulation of CD3+ T cells at the site of LA injury and at distant tumors. These findings suggest that LA treatment induces a Th1 immune response. Retention of CD3+ cells at the site of injury could be due to the presence of antigen presenting cells activated by HSP70 and displaying antigens from necrotic cells after LA treatment. While both Kupffer cells and CD3+ T accumulated at the tumor host interface and the injury site of the ablated tumor, only CD3+ T cells showed significant accumulation at the tumor host interface of distant tumors. This finding implies that a large proportion of CD3+ cells must recognise tumor specific signals, whereas KCs respond to a general inflammation response and specifically accumulate in the ablated tissues. Accumulation of CD3+ T cells within tumor margins of untreated tumors following thermal ablation treatment have also been reported in other studies using different tumor models [14,27].

In addition to local upregulation of IFNγ, significantly more splenocytes in LA treated animals produced IFNγ compared to sham treated animals indicating induction of a systemic Th1 immune response. IFNγ is produced by activated T cells and other cells of the immune system such as NK cells. The temporal kinetic pattern of IFNγ expression in this study was similar to that of CD3+ cell infiltration following LA treatment. This finding suggests that the infiltrating CD3+ T cells would also be activated along the Th1 pathway and may provide an effective mechanism for control of CRC liver metastases.

## Conclusions

We have shown LA treatment induces significant innate and adaptive immune responses, including IFNγ upregulation locally and systemically, indicating these responses to be Th1 and therefore tumor inhibiting. The accumulation of CD3+ T cells and the increase of IFNγ in distant unablated tumors suggest that the response could be beneficial in suppressing outgrowth of residual micrometastases in the clinic. Future work will identify the composition and activation status of the CD3+ T cell population after LA therapy and will validate their protective roles as their modulation may further enhance treatment outcomes.

## Competing interests

The authors declare that they have no competing interests.

## Authors' contributions

WXL carried out tissue immunostaining, ELISPOT assays, data collection, data analysis and and helped to draft the manuscript. TF participated in the study design, ELISPOT assays, data collection and data analysis and wrote the manuscript. CM-W contributed to tissue collection, performed statistical data analysis and edited the manuscript. MN participated in the study design, performed the ablations and collected tissues. VM contributed to the study design, performed some of the ablations and contributed to data analysis. LN contributed to immunostaining and data collection. CC contributed to the study design and critically revised the manuscript. All authors read and approved the final manuscript.

## Supplementary Material

Additional file 1 **Figure S1: Temporal changes in CD3+ T cells and Kupffer cells in the liver parenchyma distant from injury sites following liver LA treatment**. Groups of mice (n >5) had LA treatment in the liver and tissue was collected at various time points post ablation as indicated. Control mice had sham LA performed (LA probe was not activated), black bars = sham ablated liver, grey bars = ablated liver. Panels a, CD3+ T cells were counted from paraffin fixed liver tissue sections stained with anti-CD3+ antibody and expressed as mean number of cells/mm^2 ^± SEM, * P < 0.05 respectively. Panel b, Kupffer cells were detected with F4/80 antibody staining. Positive areas were calculated using image pro plus software. Values are expressed as mean arbitrary units ± SEM. * P < 0.05, compared to sham controls. Click here for file
